# Supplementary material for: Association of gut microbiota with depression post-myocardial infarction: A systematic evaluation and meta-analysis protocol
Source: PLoS One. 2024 Aug 9;19(8):e0305428. doi: 10.1371/journal.pone.0305428 (PMC11315350; doi:10.1371/journal.pone.0305428)
Supplement: Attachment — Submitted filename: Response to Reviewers.docx [file pone.0305428.s002.docx]

**Title: Association of intestinal flora with** **post-myocardial infarction depression: a systematic evaluation and meta-analysis protocol**

Dear editor,

Thank you very much for your feedback on the manuscript.The comments of reviewers were extremely insightful and enabled us to greatly improve the quality of our manuscript. In the following pages are our point-by-point responses to each of the comments of the reviewers. We have revised the article according to the reviewer's suggestions, in which the revised parts are in red font, while the revision process retains the traces of revision for review process.

We have meticulously revised and polished the language of the manuscript through a professional English editing service.

We hope that with these revisions and our responses, our manuscript is now suitable for publication in ***PLOS ONE*.**

We shall look forward to hearing from you at your earliest convenience.

Yours sincerely，

Xiang Liu

Beijing University of Traditional Chinese Medicine

2024.4.1

**In relation to Journal Requirements:**

When submitting your revision, I addressed these additional requirements.

1. Please ensure that your manuscript meets PLOS ONE&#39;s style requirements, including those for file naming.The PLOS ONE style templates can be found at<https://journals.plos.org/plosone/s/file?id=wjVg/PLOSOne_formatting_sample_main_body.pdf>and <https://journals.plos.org/plosone/s/file?id=ba62/PLOSOne_formatting_sample_title_authors_affiliations.pdf>

**Response:**This manuscript conforms to your journal's style requirements.

2. Thank you for stating the following financial disclosure: &quot;the National Natural Science Foundation of China (grant number No. 82174332).&quot;

Please state what role the funders took in the study.  If the funders had no role, please state: &quot;The funders had no role in study design, data collection and analysis, decision to publish, or preparation of the manuscript.&quot;If this statement is not correct you must amend it as needed.Please include this amended Role of Funder statement in your cover letter; we will change the online submission form on your behalf.

**Response:**The amended Role of Funder statement is as follows:The funders had no role in study design, data collection and analysis, decision to publish, or preparation of the manuscript.

3. Thank you for stating the following in the Acknowledgments Section of your manuscript: &quot;This research was supported by the National Natural Science Foundation of China (grant number No. 82174332).&quot;

We note that you have provided funding information that is not currently declared in your Funding Statement. However, funding information should not appear in the Acknowledgments section or other areas of your manuscript. We will only publish funding information present in the Funding Statement section of the online submission form.

Please remove any funding-related text from the manuscript and let us know how you would like to update your Funding Statement. Currently, your Funding Statement reads as follows: &quot;the National Natural Science Foundation of China (grant number No. 82174332).&quot;

Please include your amended statements within your cover letter; we will change the online submission form on your behalf.

**Response:**The funding information has been removed from the manuscript. The funding statement remains unchanged:Acknowledgments:This research was supported by the National Natural Science Foundation of China (grant number No. 82174332).

4. Please provide a complete Data Availability Statement in the submission form, ensuring you include all necessary access information or a reason for why you are unable to make your data freely accessible. If your research concerns only data provided within your submission, please write &quot;All data are in the manuscript and/or supporting information files&quot; as your Data Availability Statement.

**Response:**I modified online refer to Data Availability Statement:No datasets were generated or analysed during the current study. All relevant data from this study will be made available upon study completion.

5. Please amend the manuscript submission data (via Edit Submission) to include authors Jiqiu Hou, Weizhe Zhao, and Wanli Ding.

**Response**:The above author information has been amended online.

**To Reviewer 1**

Dear Reviewer,

Thank you for taking the time to review our manuscript and providing your constructive feedback. We sincerely appreciate your valuable comments and suggestions. In accordance with your feedback, we have revised the manuscript.The specific modifications to the content of the article are as follows.

1 Please follow the PRISMA 2020 statement.Since this review will include randomized trials and observational studies (metioned in 2.2.1 Type of study), which methods will be selected to analyze the data/evidence from these two types of clinical trials?

**Response**:Thank you for your valuable suggestion.Your question is of high significance.The statement in the text has been amended to "PRISMA 2020 Statement" and the cited references have been updated.With regard to the choice of the two types of research methods, according to my understanding, my explanation is as follows, and I am grateful to the teacher for criticising and correcting me.Although this review included two types of studies, Revan 5.3 was selected for meta-analysis. Among them, the protocol of this meta-analysis was formulated in terms of participant type,outcome indicator,search strategy,study selection,data extraction,risk of bias,and data synthesis. In addition, the selection of models was evaluated according to the level of heterogeneity.For example:We will examin the statistical heterogeneity using the I^2^ statistic. If the I^2^ value exceeds 50%, significant heterogeneity will be considered, and the random effects model will be employed. Otherwise, the fixed-effects model was used.At the same time, the programme mentions that subgroup analyses will be conducted according to the study type. Therefore, regardless of the type of studies included in this study, the meta-analysis will be conducted according to the steps in the protocol.The above is only a personal opinion, if there is something inappropriate, I hope the teacher do not hesitate to advise. Thank you again for your valuable advice.

**To Reviewer 2**

Dear Reviewer,

Thank you for your recognition of this study.Your suggestions were extremely insightful and enabled us to greatly improve the quality of our manuscript.In accordance with your suggestions, we have revised the manuscript, added much needed details,and subjected the language to rigorous editing and refinement through a professional English editing.The specific modifications to the content of the article are as follows:

**Main concerns**

1 Study designs to be included in the SR: Observational studies such as cross sectional or case-control cannot determine temporality in a relationship. One may never know if the depression preceded or came after the MI. Since the relationship that is sought in the SR is temporal, I advise the authors to consider excluding cross sectional and case control studies.

**Response:**Thanks to your valuable advice.Cross sectional and case control studies have been deleted from the text (2.2.1) as suggested.

2 Search strategy is not clear: Although listed in the text, cross-sectional design is not shown in the outlined search strategy (Table 1), so it is not clear if this design will be targeted. Furthermore, case-control is excluded, whereas in the text (under 2.2.1) it is included.

**Response:**Thank you for your important comments.Cross-sectional and case-control studies have been deleted in 2.2.1, and it has been checked that the reference in Table 1 is to case control[title/abstract] OR cross-section[title/abstract] in part 6# (7# has been labelled as the type of study to be excluded here).

3 The Chinese search terms and search strategy must be provided in an appendix.

**Response:**Thanks to your advice.Chinese search terms and search strategies have been added and uploaded in the Appendix.

4 Will duplicates be removed? From past experience, I have seen that studies published in Chinese may have their exact published duplicates although published in another language, say English. Also, the same team may numerously publish the same study. How will these be screened out?

**Response:** Duplicates will be removed and relevant changes were made in response to the literature selection issues mentioned in section 2.5 of the article:"All articles will be screened and those deemed ineligible or duplicate publications by two researchers were removed，any disagreements will be resolved by a third researcher. Details will be then recorded in an Excel spreadsheet. The following data sets will be extracted: author name, year of publication, language, study object,age,study type, sample size, age, outcome measures, interventions (if exists), controls (if exists),and adverse events."Thus this step can avoid duplication of included studies to some extent. And the joint participation of two researchers can avoid the omission of one person in entering the form and screening process. At the same time, Meanwhile, this study added the following statement to the section:"Finally, use Endnote software to remove duplicates.For duplicated publications, the study with the largest number of patients were included.

5 Data extraction (2.5), it is not clear what is meant by &ldquo;efficacy measures&rdquo; or &ldquo;complications&rdquo;. Please clarify. All study outcome measures must be listed in the data extraction sheet.

**Response:**Thanks to your valuable advice. In the Data extraction (2.5) section of the article, the words has been corrected to "outcome measures, and adverse events" due to ambiguity caused by inaccurate words(efficacy measures or complications). Outcome measures and adverse events can be found in Table 1 Search strategy for PubMed databases.

6 The analysis section needs a total revision, and some sentences need a rewrite. It is not clear what the &ldquo;binary effect size&rdquo; relates to. Please clarify and add more detail to the statistical methods.

**Response:**Thank you for such detailed advice on this section.The analysis section has been completely revised and the relevant sentences involved have been rewritten as suggested.The specific modifications are as follows:

1)Through extensive review of the literature and careful consideration, it is found that this study does not involve binary effect size, so this part has been deleted, and we apologise for any inconvenience caused.

2)What is meant by the statement &ldquo;When heterogeneity is high, we will first analyse and treat it&rdquo;. Did you mean that factors contributing to the heterogeneity will be investigated? Also please clarify how will high heterogeneity be determined.

**Response:**We are deeply grateful for your invaluable advice.High and low heterogeneity are mainly related to model selection issues, so the heterogeneity-related statements have been modified and conditions have been added regarding the judgement of high and low heterogeneity.

3)Change the tense of following sentence to future tense. &ldquo;The source of heterogeneity was assessed by sensitivity analysis, and after excluding studies of low quality or small sample sizes, the results were robust if heterogeneity did not change significantly.&rdquo;

**Response:**Thank you very much for your suggestion.The following sentences have been changed to the future tense:After excluding studies of low quality or small sample sizes, the results will be robust if heterogeneity does not change significantly.

4)The sentence &ldquo;Otherwise, excluded studies may be a source of heterogeneity&rdquo; is not clear.

**Response:**We are deeply grateful for your invaluable advice.The sentence mentioned above:” Otherwise, excluded studies may be a source of heterogeneity.”It is a further elaboration of the previous sentence, which aims to show that low quality or small sample size of studies may be a factor affecting heterogeneity, thus the previous sentence already mentioned that:”After excluding studies of low quality or small sample sizes, the results will be robust if heterogeneity does not change significantly”.This sentence, on the other hand, illustrates the converse of the above, aiming to show that if heterogeneity changes significantly after excluding low quality or small sample size of studies, the excluded studies are are are a source of heterogeneity.

5)The following sentence is not clear &ldquo;In this study, the included studies assessed using the funnel plot have fewer than 10 publications biased, otherwise Egger regression tests will be used.&rdquo;

**Response:**Thank you very for this valuable suggestions.I have modified the "In this study, the included studies assessed using the funnel plot have fewer than 10 publications biased, otherwise Egger regression tests will be used. " mentioned above,and also explained the conditions for the use of funnel plot and Egger test as shown below:Given to the limited capacity of funnel plots when pooling a small number of trials, we will use funnel plots to when the included studies fewer than 10 publications, otherwise Egger’s test will be used to verify the potential publication bias.

6)Besides depression severity, I advise the authors to conduct sub analyses by study design and by geographic region of the study.

**Response:**Thanks to the valuable advice, the research design and the geographical region of the study have been added to the sub analyses approach.

7)The planned sensitivity analysis is not clear.

**Response:**Thank you for your advice.I have added the following to the sensitivity analysis section:We will also Improve stability by changing analysis models, inclusion and exclusion criteria, or by excluding a certain type of literature.At the same time, I have revised the relevant parts of the above text on sensitivity analysis and heterogeneity.

8)How will missing data be dealt with?

**Response:**Thank you for your question.The method of handling missing data was explained in the text (section 2.5) with the addition of relevant statements, as shown below:If data is found to be missing, the author will be contacted through email or other methods to obtain the data. If there is no response, we will only analyze the existing data and describe the causes and effects of this exclusion in the paper.

7 Abstract: Study measures and methods need further clarification. The main outcome measure(s) should be stated. Also please clarify by what factors will the sub-analyses be conducted. The analysis plan is not clear. What method will be employed in running forest plots? Will the authors consider random effect models? Please clarify in the Abstract. In abstract, please remove all citations.

**Response**:We are very grateful for your invaluable advice. The main outcome measures of the study, factors for which sub analyses were conducted have been added to the abstract. The use of random effect models and conditions have been described. Also, the method of data analysis has been changed to RevMan V.5.3 which in line with section 2.7 below, and Stata has been deleted. It has been stated that the random forest plots will be carried out using RevMan V.5.3. The citations in the abstract has been removed and the citation numbers of the reference has been corrected.
