# Supplementary material for: Association of gut microbiota with depression post-myocardial infarction: A systematic evaluation and meta-analysis protocol
Source: PLoS One. 2024 Aug 9;19(8):e0305428. doi: 10.1371/journal.pone.0305428 (PMC11315350; doi:10.1371/journal.pone.0305428)
Supplement: Attachment — Submitted filename: Response to Reviewers.docx [file pone.0305428.s003.docx]

**Association of gut microbiota with** **depression post-myocardial infarction: a systematic evaluation and meta-analysis protocol**

Dear editor/reviewer,

We would like to thank you for your efforts in reviewing our manuscript, providing many helpful suggestions and guiding our research in the future. The comments were extremely insightful and enabled us to greatly improve the quality of our manuscript.

We have studied the comments point by point, revised the manuscript accordingly. The amendments are highlighted in yellow in the revised manuscript. All authors have approved the response letter and the revised version of the manuscript.

We hope that the revised version of the manuscript is now acceptable for publication in ***PLOS ONE***. If you have any queries, please do not hesitate to contact me.

Thank you again for your valuable comments and suggestions. I look forward to hearing from you soon.

Yours sincerely，

Xiang Liu,Haibin Zhao

Beijing University of Chinese Medicine

Telephone: 18810620797

Email:460259680@qq.com;haibin999@126.com

2024-5-16

**To Reviewer 1**

Dear Reviewer,

We really appreciate you for your carefulness and conscientiousness. Your suggestions are really valuable and helpful for revising and improving our paper. According to your comments, we have made the following revisions on this manuscript.

The reviewer comments are laid out below in italicized font. Our response is given in normal font and amendments or supplement to the manuscript are highlighted in yellow.

1. *Your response did not answer my previous comment "Since this review will include randomized trials and observational studies (metioned in 2.2.1 Type of study), which methods will be selected to analyze the data/evidence from these two types of clinical trials? ".*

Response：I am sorry that I did not understand the teacher's question before and apologize for not answering that question in my previous reply. As the data types of the two clinical trials are different, their analysis methods, and bias evaluation methods are different. I have discussed the methods used for the two trial types in detail in “2.6 Risk of bias (on page 6)” and “2.7 Data synthesis (on page 6-7)” of the manuscript respectively. At the same time, we have supplemented “2.2.3 Outcome measures (on page 4)” to explain the different measures that will be used in the two two types of clinical trials. And once again, I apologize for the inconvenience caused to your, and thank you very much for your patience in correcting me.

2. *The manuscript contains fundamental errors. The strategy of a model choice for meta-analysis on the heterogeneity result is wrong.*

Response: Thank you for your comments, we are very sorry for this fundamental error. According to your suggestions, we have revised the model selection strategy for the meta-analysis after careful reading of a variety of methodological articles, the revised results of which are shown in “2.7 Data synthesis (on page 7)”.

3. *How to assess the quality of observational studies?*

Response: Thank you for your suggestion. After consideration, we planned to assess the quality of observational studies through RoBANS. The modifications in “2.6 risk of bias (on page 6)”.

4. *An appealing review should include a critical assessment of the relevant literature published.*

Response: Thank you very much for your valuable comments and suggestions. Due to the fact that different studies require different treatment assessments. Therefore, we will apply the Cochrane to evaluate the quality of the interventional trials. The quality of the observational study will be evaluated independently by the Risk of Bias Assessment Tool for Nonrandomized Studies (RoBANS). This section is described in “2.6 Risk of bias (on page 6)” in the text.

Again, thank you for giving us the opportunity to strengthen our manuscript with your valuable comments and queries. We have worked hard to incorporate your feedback and hope that these revisions persuade you to accept our submission.

**To Reviewer 2**

Dear Reviewer,

We feel great grateful for your professional review work on our article. In accordance with your suggestions, we have made the following revisions to our manuscript. Our response is given in normal font, and modification or supplement to the manuscript are highlighted in yellow.

*1. The whole section under “2.7 Data synthesis” needs major revision. It appears that sections were taken from other published sources without them being properly edited or adapted to this study. The tenses used must all be in future tense and not past tense.*

Response: Thanks for the valuable advice, I have completely revised the section and changed the tense to future tense in “2.7 Data synthesis (on page 6-7)”.

*2. Some sentences still do not make sense and must be revised. For example, the following sentence is not clear:
“Given to the limited capacity of funnel plots when pooling a small number of trials, we will use funnel plots to when the included studies fewer than 10 publications, otherwise Egger’s test will be used to verify the potential publication bias.”*

Response: Thanks to your reminder, considering the limitation of the funnel plot (If the number of studies is less than 10, it will be difficult to find the cause of the asymmetry), this study was planned to use Egger’s test entirety to examine potential publication bias in “the last line of 2.7 Data synthesis (on page 7)”.

*3. Since the depression scales are continuous, it is not clear “Odds Ratios” of what will be estimated? Also, it is not clear mean difference of what measures will be estimated? I am assuming that the mean differences in depression scale scores between those who did and did not have a myocardial infarction will be estimated. This needs to be clearly stated.*

Response: Thank you for your advice, we benefited a lot. Inspired by your comments, we consider the importance of gut microbiota in the text, previously in the “Data synthesis” did not expand the interpretation of observational studies about gut microbiota, so in addition to the depression scale, we supplemented the indicators related to gut microbiota, which can be reflected in the “2.2.3 outcome measures (on Page 4)”, for the methodological differences between different research methods are reflected in the “2.7 Data synthesis (on Page 6)”. For the description of MACE, it is more appropriate to use RR values, so we deleted the Odds Ratios. Thank you again for your positive comments and valuable suggestions to improve the quality of our manuscript.

*4. The decision to run random effect models when the heterogeneity is 50% or more is a common methodological mistake that authors do. A non-significant heterogeneity test does not necessarily indicate that the studies share a common effect size. Sometimes the test of heterogeneity is underpowered and therefore can provide non-significant results even when there is heterogeneity.
The choice of a model should be based on the question of which model fits the distribution of effect sizes. The random effects model is often the more careful and credible approach that is used when meta-analysing results from different studies.*

Response: Thank you for your patience and advice. We have learnt a lot from the reference. Your suggestions are important for improving the quality of this manuscript. I have made modifications for model selection in “2.7 Data synthesis (on Page 7)”.

We deeply appreciate your valuable comments and suggestions, and look forward to hearing from you regarding our submission. We would be glad to respond to any further questions and comments that you may have.

**To Reviewer 3**

Dear Reviewer,

We feel great thanks for your professional review work on our article. According to your comments, we have made extensive modifications to our manuscript point to point. In accordance with your suggestions, we have made the following revisions to our manuscript. Our response is given in normal font and changes or additions to the manuscript are highlighted in yellow. In addition, in order to comply with the word count requirements in the guidelines, we streamlined the text, and the deleted traces was retained for viewing.

*1. Following the recommendations of the previous reviewers, I suggest the following enhancements: The term "flora" is outdated and should be replaced with "microbiota."*

Response: We sincerely thank the reviewer for careful reading. As suggested by the reviewer, we have corrected “flora” into “microbiota”. The modifications are highlighted in yellow.

*2. Utilize abbreviations like GBA for gut-brain axis and other necessary terms.*

Response: Thanks for your careful checks. Abbreviations have been made throughout the text, such as GBA, MI, CAD.

*3. Trim the abstract length in accordance with the journal's guidelines.*

Response: Abstract lengths have been trimmed according to journal guidelines. Our response is given in normal font and changes or additions to the manuscript are highlighted in yellow. In addition, in order to control the word count, we streamlined the text, and the deleted traces retained for reviewing.

4. *Improve the writing; consider employing an AI tool to streamline ideas and enhance syntax.*

Response: Thanks for your suggestion. With tools such as AI, we tried our best to modify and improve the manuscript. The modifications are highlighted in yellow.

5. *Enhance the quality and/or resolution of the images.*

Response: Thank you for your valuable suggestion. We have improved the image quality. I have uploaded the original image with an attachment in system to prevent the image from affecting the resolution after inserting into the manuscript.

6. *Table 1 lacks substantive content; I propose integrating its description into the text.*

Response: We sincerely appreciate the valuable comments. According to the suggestion, we have incorporated the search strategy for the keywords mentioned in Table 1 into the text. Due to word limitations, it was difficult to incorporate all the content in the table into the text, so the keywords in the text have been streamlined. At the same time, in order not to take up more words, we have replaced table 1 with an image to be inserted into the text, as shown in “2.3 Search Strategy (on Page 4-5)”.

7. *Consider including a concise visual abstract if feasible.*

Response: We think this is an excellent suggestion. However, given the partial duplication between the visual summary and Figure 1, we did not choose to use the visual summary for further presentation. We have simplified the abstract to make it clearer. The modifications are visible at the “Abstract (on Page 1-2)”.

8. *Overall, it's a promising piece of work, but refinement is needed before publication.*

Response: Thank you again for your constructive comments and suggestions on our manuscript. We hope you will find our revised manuscript acceptable for publication.
